# Specific MRP4 Inhibitor Ceefourin-1 Enhances Apoptosis Induced by 6-Mercaptopurine in Jurkat Leukemic Cells, but Not in Normal Lymphoblast Cell Line CRL-1991

**DOI:** 10.3390/medicina58060695

**Published:** 2022-05-24

**Authors:** Edgardo Becerra, Laura Berumen, Valeria Soto-Ontiveros, Guadalupe García-Alcocer

**Affiliations:** 1 Posgrado en Ciencias Químico Biológicas, Facultad de Química, Universidad Autónoma de Querétaro, Cerro de las Campanas S/N, Querétaro 76010, Mexico; ebecerra1989@gmail.com (E.B.); lcbsq@yahoo.com (L.B.); 2 Facultad de Química, Universidad Autónoma de Querétaro, Centro Universitario, Querétaro 76010, Mexico; valeria.soto.ontiveros@gmail.com

**Keywords:** 6-mercaptopurine, MRP4, leukemia, ceefourin-1, MRP4 inhibitor, apoptosis, cell proliferation

## Abstract

*Background and objectives*: The multidrug resistance protein 4 (MRP4) is a member of the ABC transporter, which has been extensively related to many types of cancer including leukemia. MRP4 overexpression and activity over the efflux of some chemotherapeutic drugs are the main causes of chemoresistance. 6-mercaptopurine (6-MP) is a chemotherapeutic drug widely used in the consolidation and maintenance phases of leukemia treatment. However, 6-MP is a substrate of MRP4, which decreases its chemotherapeutic efficacy. Current research is focused on the development of MRP4 inhibitors to combat chemoresistance by allowing the accumulation of the drug substrates inside the cells. To date, the only specific MRP4 inhibitor that has been developed is ceefourin-1, which has been reported to inhibit MRP4 in many cancer cells and which makes it an excellent candidate to enhance the activity of 6-MP in a combined treatment in vitro of leukemic cells. *Materials and methods:* in the present work, we determined the enhancing activity of ceefourin-1 on the antiproliferative and apoptotic effect of 6-MP in leukemic Jurkat cells by trypan blue assay and flow cytometry. Besides, we determined the 6-MP and ceefourin-1 binding sites into MRP4 by molecular docking and molecular dynamics. *Results:* ceefourin-1 enhanced the apoptotic activity of 6-MP in Jurkat cells, while in CRL-1991 cells both antiproliferative and apoptotic effect were significantly lower. Ceefourin-1 additively cooperates with 6-MP to induce apoptosis in leukemic cells, but normal lymphoblast CRl-1991 showed resistance to both drugs. *Conclusion:* ceefourin-1 and 6-MP cooperates to trigger apoptosis in leukemic Jurkat cells, but the full mechanism needs to be elucidated in further works. In addition, our perspective is to test the cooperation between ceefourin-1 and 6-MP in samples from patients and healthy donnors.

## 1. Introduction

Multidrug resistance protein 4 (MRP4) is encoded by the ABCC4 gene located on chromosome 13. MRP4 transports mainly organic anions and glucuronide conjugates [1]. Since MRP4 is present in multiple organs, the cellular exposure to drugs or metabolites can be modified leading to metabolic dysregulation. The MRP4 expression in most cancer cells is pivotal for the treatment because the protein can efflux many anticancer drugs or interfere with physiopathological processes.

The relation between MRP4 overexpression in 6-mercaptopurine (6-MP) resistance was reported in T-lymphoblastic leukemia (T-ALL) cells (CEM cells), where MRP4 was up-regulated in tumorous cells but influx transporters were down-regulated, leading to a high efflux of 6-MP and survival of resistant cells [2]. In addition, the overexpression of both MRP4 and MRP1 are reported to be significantly associated with poor prognosis in neuroblastoma [3]. On the other hand, MRP4 is the main regulator of the intracellular (icAMP) levels in human leukemia cells, promoting its efflux. Furthermore, the inhibition of MRP4 is associated with an increase in icAMP, leading to lower proliferation, promotion of cell differentiation, and intrinsic apoptosis activation [4,5]. The inhibition of MRP4 expression with shRNA inhibits cell growth and increases the percentage of cells in G1 phase to further apoptosis, which set MRP4 inhibition as a novel therapeutic strategy in leukemia [6].

The effect of icAMP elevation can be pro-apoptotic or anti-apoptotic, depending on the cell type. In leukemias/lymphomas, cAMP elevation is reported to be pro-apoptotic, whereas in normal blood cells, it is reported to be anti-apoptotic [5]. According to this statement, the inhibition of cAMP efflux by targeting MRP4 should be sufficient to selectively trigger apoptosis in cells that depend on cAMP efflux as an anti-apoptotic mechanism for survival.

Nowadays, the pharmacological inhibition of MRP4 is limited due to the absence of specific small molecule inhibitors (SMIs) of MRP4. The SMIs dipyridamole and indomethacin also target other related ABC transporter family members [7,8]. MK-571, the most widely SMI used to inhibit MRP4 also blocks MRP1, MRP2, MRP3, and MRP5 [8,9], the uptake transporter OAT1B1/SLCO1B1 [10], antagonizes cysteinyl leukotriene receptor 1 and inhibits phosphodiesterases (PDEs) [11]. Ceefourin-1 is a benzothiazole-containing compound whose selectively inhibits MRP4 over other ABC transporters, which was determined by high-throughput screening [12]. Its selective MRP4 inhibition activity has been recently reported, and it is not used in any chemotherapy protocol. With the aim to establish the effects of MRP4 inhibition in a cancer cell line, we studied the physiological effect of the specific MRP4 inhibitor ceefourin-1.

The low cellular toxicity and high stability of ceefourin-1 is well documented, and it is a promising molecule to reverse chemoresistance over those anti-cancer drugs that are substrates of MRP4. Moreover, MRP4 contains multiple substrate binding sites [13,14], thus, inhibitors may differentially affect the transport of different substrates [15]. Since ceefourin-1 blocks the transport of structurally diverse MRP4 substrates as SN-38 (an irinotecan metabolite and topoisomerase 1 inhibitor [3]), E217bG, D-luciferin, cAMP, and 6-MP [12], we studied the antiproliferative and apoptotic effect of ceefourin-1 alone and in combination with 6-MP in both Jurkat T-ALL cells and normal lymphoblasts (CRL-1991) to determine if ceefourin-1 enhances the chemotherapeutic activity of 6-MP.

## 2. Materials and Methods

### 2.1. Drugs

To perform apoptosis and proliferation assays, both molecules 6-MP (ab142389) and ceefourin-1 (Cat number ab145144) were obtained from ABCAM, with a purity > 98%.

### 2.2. Cell Culture

Jurkat cells (ATCC, No. TIB-152) and T1 (174 x CEM.T1) (ATCC CRL-1991) were obtained from the American Type Culture Collection (ATCC, Manassas, VA, USA) and were grown at 37 °C in a humidified 95% air/5% CO_2_ atmosphere. Jurkat cells were cultured in RPMI 1640 medium (ATCC, No. 30-2001), and CRL-1991 cells were cultured in Iscove’s modified Dubelcco´s medium, both mediums containing 10% fetal bovine serum (FBS, ATCC, No. 30-2020) and antibiotics (penicillin and streptomycin sulphate).

### 2.3. Proliferation Assay and Viability

To determine the effect of ceefourin-1 and 6-MP over the cellular proliferation and viability, 1 × 10^6^ cells/mL were treated with the concentrations listed in Table 1 and counted after 24 h. The cell counting was performed in a CytoSmart cell counter, cloud-based image processing to perform cell counting from Corning (Cat number 6749), following the trypan blue protocol [16].

### 2.4. Apoptosis Assay

The apoptosis assay was performed with the FITC Annexin V Apoptosis Detection Kit I (No. 556547), according to the manufacturer’s instructions. Briefly, 1 × 10^6^ cells were treated with different 6-MP and ceefourin-1 concentrations of the molecules (Table 1). After 24 h of drug treatment, the cells were washed twice with cold phosphate-buffered saline (PBS) and then resuspended in binding buffer at a concentration of 1 × 10^6^ cells/mL. An amount of 100 μL (1 × 10^5^ cells) was transferred to a 5 mL culture tube, and the cells were then stained with 5 μL of FITC Annexin V and 5 μL of propidium iodide. The cell suspension was gently mixed with a vortex and incubated for 15 min at room temperature. After incubation, 400 μL of 1× Binding Buffer was added to each tube, and the cells were analyzed by flow cytometry in a BD FACSVerse^TM^ system (BD Biosciences, MH, México city, México) and BD FACSuite^TM^ software, within 1 h.

Data were normalized to the maximum signal, and concentration–response curves were fitted with a three-parameter logistic model by non-linear regression using Prism 8 to determine the concentration that inhibits 20% and 40% of proliferation and the concentration that induces 20% and 40% of cell death. To determine significant differences between groups are represented as different letters (*p*-value ≤ 0.05), according to two-way ANOVA and Bonferroni´s multiple comparison test.

### 2.5. Molecular Docking

To study the binding site and the intermolecular interactions of 6-MP and ceefourin-1 in MRP4, molecular docking studies were carried out using the AUTODOCK 4.2.6 software (The Scripps Research Institute, San Diego, CA, USA) [17]. The MRP4 structure, previously modeled [18], was used as an input for the AUTOGRID 4.2.6. The maps were calculated with 0.375 Å spacing between grid points. The center of the grid box was defined as follows: [−0.65 8.78 28.26], corresponding to Glu103, Gly359, Arg362, and Ser328 according to previous reports [19]. The dimensions of the active site box were set at 40 × 60 × 80 points. Rigid-flexible docking was performed for the compounds and each docked system was built using AUTODOCK, with 25 runs using Lamarckian genetic algorithm with 5 million maximum energy evaluations per run. to determine the differences in the intermolecular interactions to MRP4 related to chemical structure between each group.

### 2.6. Umbrella Sampling

In order to determine the binding energy of ceefourin-1 and 6-MP into MRP4, umbrella sampling simulations were performed by using the MRP4 C1 of the previous 25 ns AA-MD trajectory [18], the system was built under the previously mentioned conditions. Once the system was obtained and relaxed using the AA-MD relaxation protocol, a 10 ns (100 frames) MD simulation was performed in the Metadynamics module of Desmond using the protein and ligand center of mass distance as the collective variable, with 0.3 kcal/mol height and 0.1 kcal/mol width as the Gaussian parameters for the umbrella protocol, on an NPT ensemble at 310.15 K and 1.01325 bar. Finally, the analysis was performed in the Metadynamics Analysis module of Desmond.

## 3. Results

### 3.1. Effect of Ceefourin-1 and 6-MP on the Cellular Proliferation

**Figure 1:**
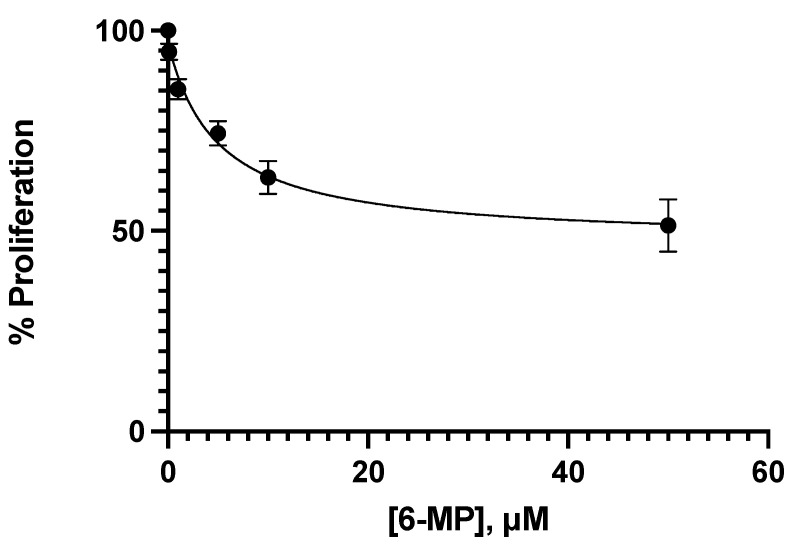
Concentration–response of 6-MP tested in Jurkat cells to study the effect on the cell proliferation at 24 h of treatment. Mean +/− SE are shown in each point.

**Figure 2:**
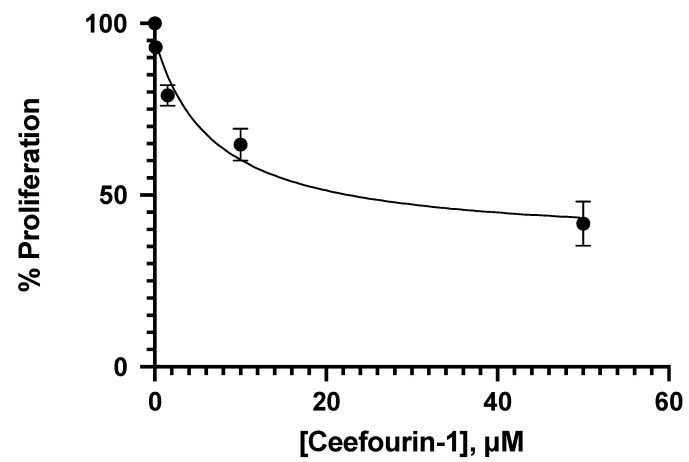
Concentration–response of ceefourin-1 tested over the Jurkat cell proliferation at 24 h of treatment. Mean +/− SE are shown in each point.

Different concentrations of 6-MP were tested to determine the concentrations that inhibit 20% and 40% of proliferation at 24 h in Jurkat cells. Such concentrations for 6-MP were 4.25 μM and 8.5 μM, respectively. The analyst error was avoided by using the automatic cell counter CytoSmart by Corning, to obtain better results. As observed in Figure 1, the effect of 6-MP over the Jurkat cells proliferation is concentration-dependent, but not linear. Trypan blue was used for the cell counting, so, the cells observed could be alive or dead, according to the principle of the proliferation test. As is shown in Figure 1, all the 6-MP concentrations impact Jurkat proliferation, but at this point, we did not determine the mechanisms involved in such an effect. In addition, four concentrations of ceefourin-1 were also tested in Jurkat cells to determine if this MRP4 inhibitor interferes with cellular proliferation to further evaluation in combination with 6-MP. As can be appreciated in Figure 2, the anti-proliferative effect of ceefourin-1 is concentration-dependent, as well as 6-MP, and all the concentrations tested interfere with Jurkat proliferation. The concentrations of ceefourin-1 (Ceef1) that inhibit 20% and 40% of proliferation were 1.5 μM and 12 μM, respectively.

**Figure 3:**
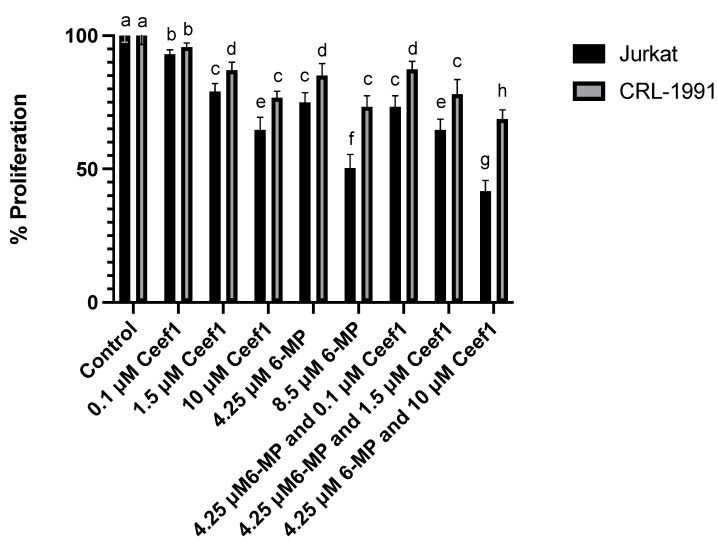
Different combinations and individual treatments of 6-MP and Ceef1 in both, Jurkat cells and CRL-1991 cells, to test their effect on cell proliferation. Mean +/− SE are shown in each column. Significant differences between groups are represented as different letters (*p*-value ≤ 0.05), according to two-way ANOVA and Bonferroni´s multiple comparison test.

In order to determine if Ceef1 cooperates or enhances the 6-MP activity over the proliferation of Jurkat cells in vitro, we treated the cells with different combinations of 6-MP and Ceef1, as indicated in Figure 3. The concentration 0.1 μM of Ceef1 induced slightly but significant higher antiproliferative effect with respect to negative control, while 1.5 μM of Ceef1 represents the 50% of MRP4 inhibition as reported by Fusaku Usuki and coworkers [20]. We decided to test 1.5 μM Ceef1 to determine if such a concentration enhances the antiproliferative and apoptotic effect of 6-MP. On the other hand, we also tested the same 6-MP and Ceef1 combinations in CRL-1991 cells with the aim of studying if both cell lines present different responses to the treatments, considering that they are metabolic and genetically different, even though they are derived from the same lineage. In Jurkat cells, all the treatments significantly reduced cell proliferation compared to the control group, where the combined treatment 4.25 μM 6-MP + 0.1 μM Ceef1 reduced cell proliferation with no significant difference with respect to 4.25 μM 6-MP alone, indicating that Ceef1 at 0.1 μM did not enhance the effect of 6-MP, at least at 24 h of treatment. The combined treatment 4.25 μM 6-MP + 1.5 μM Ceef1 significantly reduces cell proliferation compared to 4.25 μM 6-MP alone. In addition, 4.25 μM 6-MP + 10 μM Ceef1 had an additive effect at reducing cell proliferation in the same way that the sum of the individual effects of 4.25 μM 6-MP and 10 μM Ceef1. Moreover, 4.25 μM 6-MP + 10 μM Ceef1 significantly reduced cell proliferation greater than 8.5 μM 6-MP, which represents a higher concentration and possibly toxic effects.

All the treatments tested on CRL-1991 cells induced a lower inhibition of cell proliferation compared to that observed in Jurkat cells, which indicates that, theoretically, the proliferation of normal cells in a living organism would not be affected in the same manner than cancer cells. Statistically, the combined treatment of 4.25 μM 6-MP + 0.1 μM Ceef1, and 4.25 μM 6-MP alone had the same effect on the inhibition of cell proliferation. In the same way, the treatments 8.5 μM 6-MP and 4.25 μM 6-MP + 1.5 μM Ceef1 had the same effect on the inhibition of CRL-1991 cells proliferation, but 8.5 μM 6-MP induced higher inhibition on Jurkat cells proliferation compared to 4.25 μM 6-MP + 1.5 μM Ceef1. The combined treatment 4.25 μM 6-MP + 10 μM Ceef1 exerted the highest inhibition on cell proliferation in both cell lines.

### 3.2. Apoptotic Interaction between Ceefourin-1 and 6-MP

**Figure 4:**
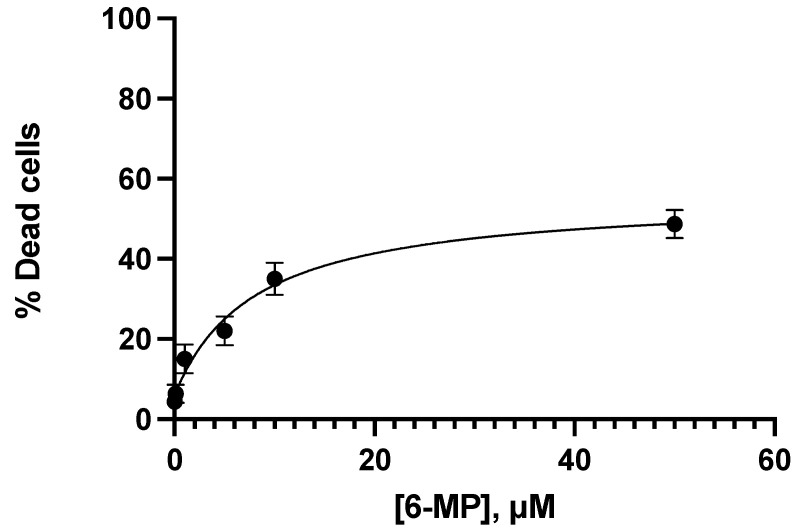
Concentration–response of different concentrations of 6-MP in Jurkat cells at 24 h of treatment to test cell viability. Mean +/− SE are shown in each point. Percentage of dead cells were identified by trypan blue.

The evaluation of the viability induction by 6-MP and Ceef1 was carried out in the same way as the proliferation assay: with the same time, same concentrations, and same cell lines, and 6.75 μM 6-MP and 13.5 μM 6-MP induced cell death around 20% and 40%, respectively. In addition, 1.5 μM Ceef1 and 12.5 μM Ceef1 induced cell death around 20% and 40%, respectively. The graph in Figure 4 indicated that the effect of 6-MP on cell death induction was concentration-dependent, being the concentration 0.1 μM statistically same with respect to the negative control. The minimum concentration of 6-MP required to induce apoptosis significantly different with respect to the negative control was 1.0 μM. It was observed that the concentrations of 6-MP and Ceef1 required to induce both cell death and inhibition of cell proliferation were quite similar.

**Figure 5:**
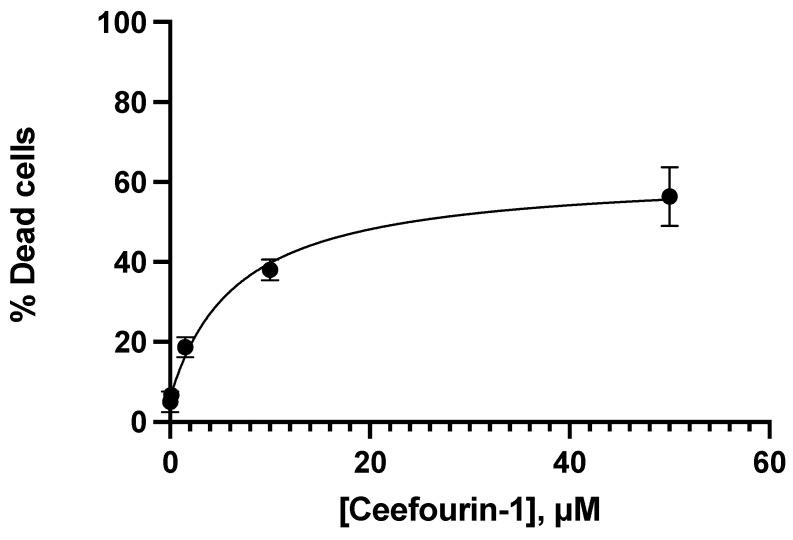
Concentration–response of different concentrations of Ceef1 in Jurkat cells at 24 h of treatment to test cell viability. Mean +/− SE are shown in each point. Percentage of dead cells were identified by trypan blue.

Ceef1 induced cell death in a concentration-dependent manner (Figure 5). The concentration 0.1 μM of Ceef1 induced the same percentage of cell death as the negative control, and the 1.5 μM concentration was the minimum concentration required to induce cell death, which is significantly different with respect to the negative control. Ceef1 50 μM induced cell death around 56%, which was 8% higher than 6-MP at the same concentration.

**Figure 6:**
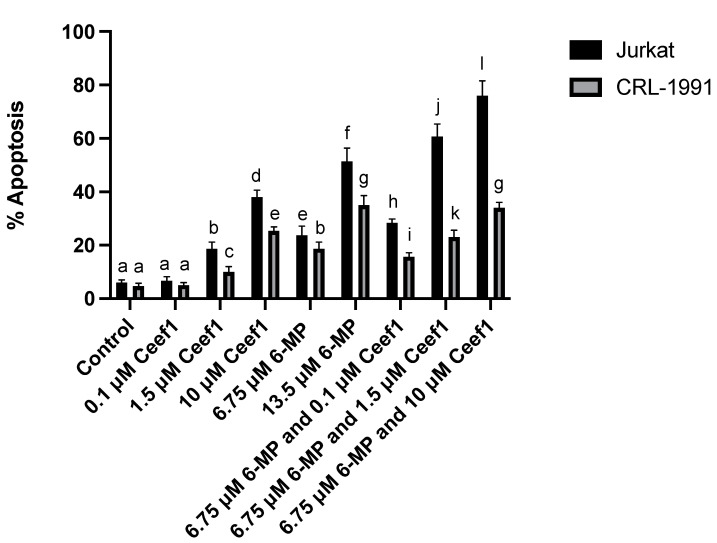
Individual treatments of 6-MP and Ceef1 and their respective combinations in both, Jurkat cells and CRL-1991 cells to test apoptosis induction. Mean +/− SE are shown in each column. Significant differences between groups are represented as different letters (*p*-value ≤ 0.05), according to two-way ANOVA and Bonferroni´s multiple comparison test.

To test if Ceef1 enhances the apoptotic effect by 6-MP, the Jurkat cells were treated as indicated in Figure 6. All the treatments with 6-MP, Ceef1, and combined significantly induced a higher percentage of apoptosis than the negative control, and in all the treatments significant differences were observed. Even though 0.1 μM Ceef1 did not induce apoptosis greater than the negative control, it enhances the apoptotic effect of 6.75 μM 6-MP. Moreover, 1.5 μM Ceef1 together with 6.75 μM 6-MP induced apoptosis 2.5 higher than 6.75 μM 6-MP and significantly higher than 13.5 μM 6-MP. It could be suggested that 6.75 μM 6-MP together with 1.5 μM Ceef1 exert synergistic cooperation rather than additive cooperation at inducing apoptosis. In addition, 6.75 μM 6-MP together with 10 μM Ceef1 induced the highest apoptotic percentage at around 75%. On the other hand, the same 6-MP and Ceef1 treatments significantly induced lower apoptosis in CRL-1991 cells than those observed in Jurkat cells. The combination of 6.75 μM 6-MP and 0.1 μM Ceef1 significantly induced lower apoptosis than 6.75 μM 6-MP alone, and 13.5 μM 6-MP significantly induced higher apoptosis than 6.75 μM 6-MP combined with 1.5 μM Ceef1. The combination of 6.75 μM 6-MP with 10 μM Ceef1 promoted apoptosis of CRL-1991 cells, similar than 13.5 μM 6-MP alone, which indicates that Ceef1 does not enhance apoptosis in the significant way it does for Jurkat cells.

The comparison of the apoptotic effect induced by each treatment in both cell lines indicated that 6-MP and Ceef1 alone or in combination, significantly induced lower apoptosis on CRL-1991 cells compared with Jurkat cells, which suggests that both molecules exerted cytotoxic effects in leukemic cells greater than in normal cells, at least in vitro. The 6.75 μM 6-MP combined with 1.5 μM Ceef1 and 6.75 μM 6-MP combined with 10 μM Ceef1 induced apoptosis higher in Jurkat cells than in CRL-1991 cells.

### 3.3. Molecular Docking and Umbrella Sampling

**Figure 7:**
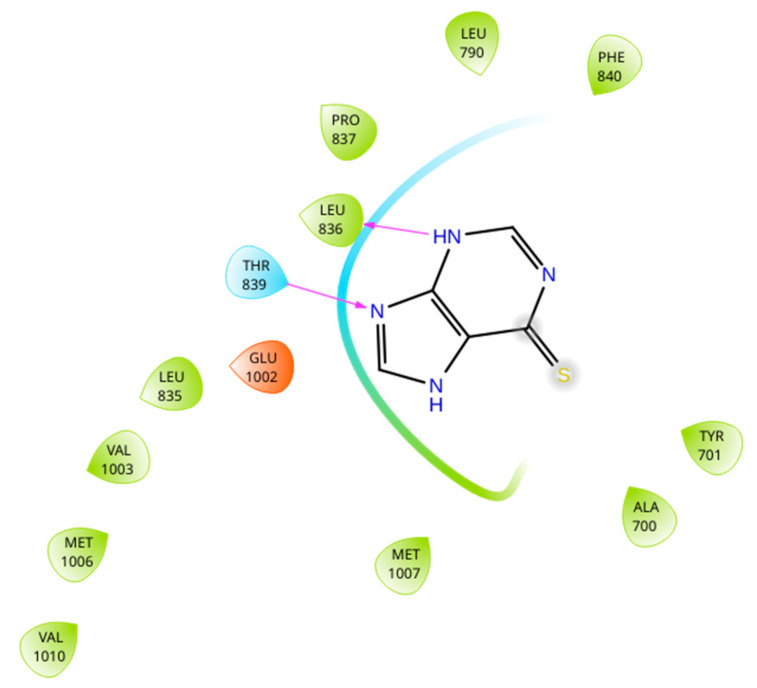
Ligand interaction diagram of 6-MP into MRP4 performed in Maestro. Green: hydrophobic interaction. Blue: polar interaction. Orange: interaction with negative charged residue. Pink arrow: H-bond.

**Figure 8:**
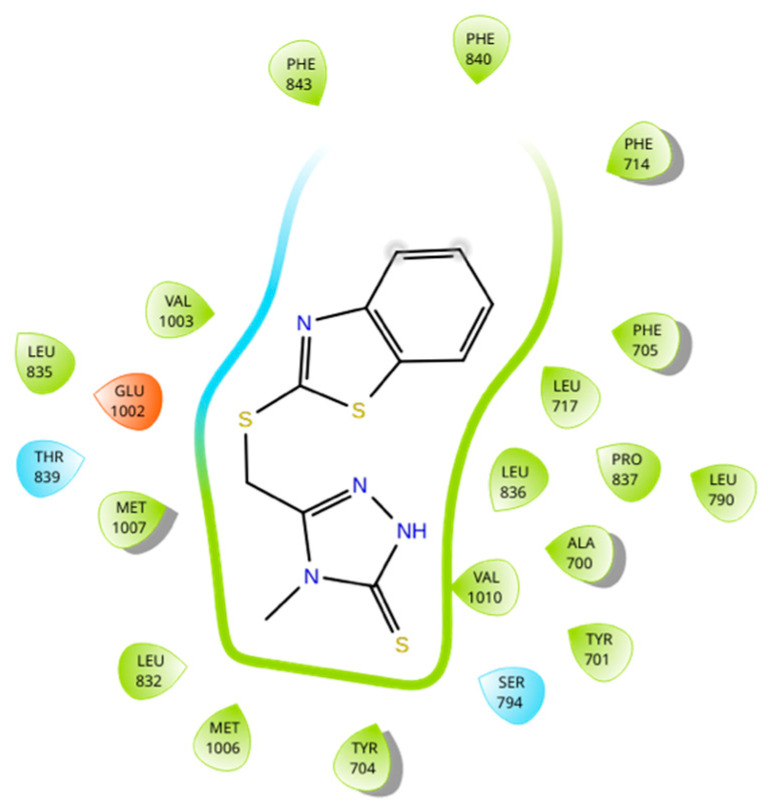
Ligand interaction diagram of Ceef1 into MRP4 performed in Maestro. Blue: polar interaction. Orange: interaction with negative charged residue.

The binding sites of MRP4 substrates and inhibitors may be located at different regions of the transmembrane domains (TMDs) of the protein. Based on the previous statement, molecular docking simulations were performed between 6-MP-MRP4 and Ceef1-MRP4 complexes, to determine if both molecules share a similar binding site. In Figure 7 the ligand interaction diagram (LID) of 6-MP into MRP4 is shown. 6-MP exerts H-bonds interactions with leucine 836 and threonine 839 into a hydrophobic pocket binding where 6-MP did not perfectly fit. Interestingly, Ceef1 interacts in the same binding site as 6-MP but did not exert H-bonds (Figure 8). Nevertheless, Ceef1 seems to perfectly fit in its hydrophobic pocket binding, where its interactions are mainly hydrophobic, and those lead to the lower binding energy. The Docking Scores (DSs) for 6-MP and Ceef1 were −4.79 kcal/mol and −7.63 kcal/mol, respectively (Table 2). The DS indicated that Ceef1 conformations require lower energy than 6-MP conformations to interact and bind to MRP4.

**Figure 9:**
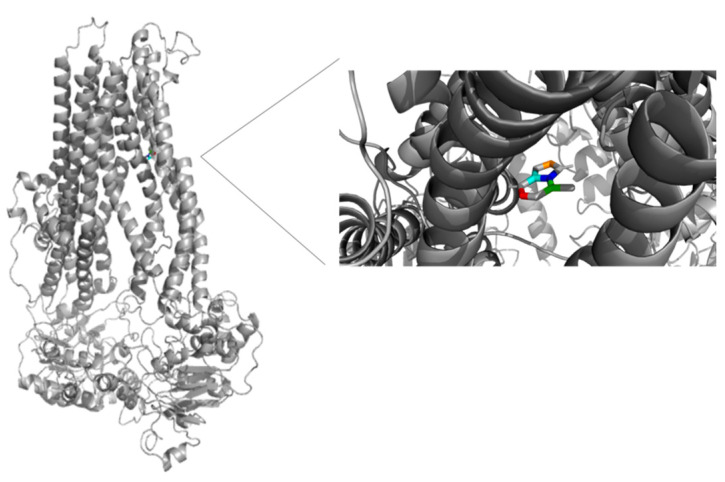
Representation of the full MRP4 structure in complex with 6-MP, and a close up to the 6-MP binding site. 6-MP is represented by the rainbow-colored structure.

**Figure 10:**
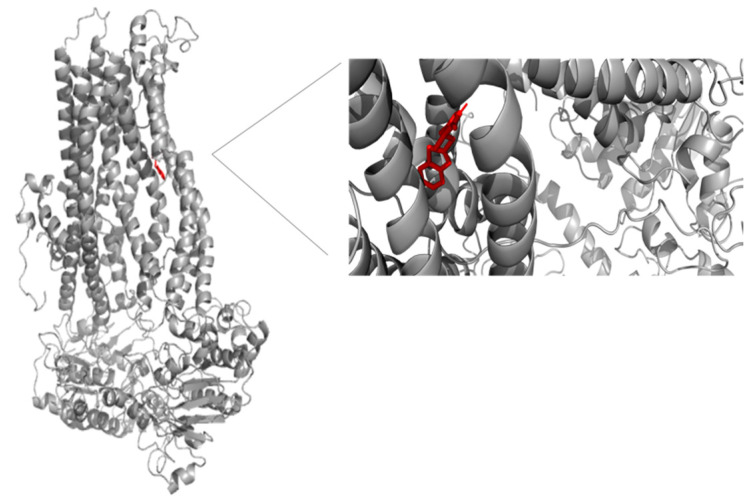
Representation of the full MRP4 structure in complex with Ceef1, and a close up to the 6-MP binding site. Ceef1 is represented by the red structure.

Figure 9 and Figure 10 show the binding sites of 6-MP and Ceef1, respectively. For both molecules the binding site is located in the TMD2, close to the inner cavity. The structure of Ceef1 is bigger than the 6-MP structure and interacts with more residues fitting better in the hydrophobic binding site. For the proximity of both 6-MP and Ceef1 binding sites into MRP4, umbrella sampling simulations were performed to determine their theoretical affinity to MRP4, expressed as free binding energy (ΔG), and according to the results presented in Table 2, Ceef1 has significantly higher affinity than 6-MP. Such results suggested that the Ceef1-MRP4 complex is more stable than the 6-MP-MRP4 complex, which is interesting regarding the efflux by MRP4 considering that 6-MP and Ceef1 would be in the cell at the same time.

## 4. Discussion

The combination of two or more drugs has been used to treat cancer for many years, and it represents both the first line of chemotherapy and the main source of drug-derived toxicity [21]. The design of combined chemotherapy relies on the pharmacological activity of the drugs, where their action must be complementary to each other—drug A enhances the action of drug B. In the present work, the antiproliferative and apoptotic effect of Ceef1 was tested in both Jurkat cells and CRL-1991 cells to further determine if it enhances the action of the 6-MP.

The concentration 0.1 μM of 6-MP was not able to interfere with the proliferation nor the significant apoptosis induction on Jurkat cells. Such results are consistent with previous reports, and no researcher has reported antiproliferative nor apoptotic effect at concentrations lower than 1 μM [12,22]. In addition, 6-MP is metabolized into their active metabolites also called tioguanine nucleotides (TGN), which are also substrates of MRP4 [23] leading to an even lower concentration of active metabolites than 0.1 μM. Any 6-MP concentration once inside the cell will be decreased by MRP4 activity. Nevertheless, it was observed that at least 1 μM of 6-MP is necessary to significantly interfere with cell proliferation, taking in consideration that a fraction of 6-MP has been previously metabolized and effluxed. Even though the antiproliferative effect of 6-MP is concentration-dependent, it is not linear because the more intracellular levels of 6-MP the more activity of metabolizing enzymes and MRP4 would be [24]. 6-MP acts by numerous mechanisms to induce antiproliferative and apoptotic effects, which includes its incorporation into the DNA chains promoting cell cycle arrest and further apoptosis. Additionally, 6-MP inhibits de novo purine synthesis, which is more important for lymphocyte proliferation than the salvage pathway [25], and there is evidence suggesting that thiopurine drugs might regulate metabolic checkpoints that promote reprogramming in normal and leukemic T cells as Jurkat cells. Such checkpoints include mTOR, AMP-activated kinase (AMPK), Myc, and HIF-1α [26], and those previous actions of 6-MP require higher concentration than 0.1 μM to get significant effects over cell proliferation and apoptosis.

In this line, Ceef1 was tested at various concentrations to determine if its previous tested ability to inhibit MRP4 also induces apoptosis or interferes with cellular proliferation [12]. In concordance with 6-MP, Ceef1 at 0.1 μM did not induce a significant antiproliferative effect on Jurkat cells, possibly because such concentration is not sufficient to regulate the activity of MRP4. The main action attributed to Ceef1, a benzothiazole-containing compound, is the selective inhibition of MRP4 among all the ABC transporters. However, the benzothiazole derivatives are known by their anticancer, anti-inflammatory, antiviral, anticonvulsant, antidiabetic, and more activities [27], which means that Ceef1 interacts with other targets, but the antiproliferative effect was dimly observed at 0.1 μM. Moreover, the more Ceef1 concentration was increased the more significant antiproliferative effect was observed, but not linearly. In the same manner, the apoptotic by Ceef1 was concentration-dependent and it was significant from 1.5 μM to 50 μM. According to previous evidence, the inhibition of MRP4 leads to increased cAMP levels to further intrinsic apoptosis activation once cAMP activated PKA [5,28], being this the most probable mechanism by which Ceef1 induces apoptosis. Nevertheless, the main question is how Ceef1 enhanced 6-MP-induced apoptosis. If 6-MP is an MRP4 substrate, and its intracellular concentration depends on the MRP4 activity, the most adequate explanation of the enhanced action of 6-MP would be that Ceef1 inhibits MRP4, and thus, blocking the efflux of 6-MP. When we treated the cells with 13.5 μM 6-MP alone, a certain amount of 6-MP must be effluxed by MRP4, but once we added Ceef1, a lower amount of 6-MP could be effluxed leading to more bioavailability inside the cell. The measure of intracellular 6-MP must be performed to demonstrate our hypothesis.

Regarding the previous statement, the more Ceef1 levels inside the cell the more levels of 6-MP and their active TGN metabolites would be bioavailable, due to the inhibition of MRP4 by Ceef1. However, is MRP4 really inhibited or how Ceef1 regulate its activity? A theoretical explanation of the inhibitory activity of Ceef1 can be addressed through the molecular docking and umbrella sampling simulations performed in this work. The molecular docking simulations indicated that both Ceef1 and 6-MP share the same binding site into MRP4, and it can be inferred that they compete for such binding site, where Ceef1 fits better than 6-MP. However, the free binding energy of Ceef1 was significantly lower than the free binding energy of 6-MP, which indicates that Ceef1 is a competitive antagonist that interferes with the binding of 6-MP into MRP4.

Furthermore, 6-MP can indirectly regulate MRP4 activity by depleting ATP. It has been determined that 6-MP induces rapid ATP depletion in Jurkat cells, even though the biochemical basis for such mechanism remains unestablished [26], and such ATP depletion may affect the function of MRP4 due to it requires ATP to start the transport cycle [29]. ATP depletion promotes metabolic stress, and it could be a possible mechanism to inhibit cell proliferation and perhaps apoptosis. Such metabolic stress might be higher in Jurkat cells because they have high intracellular ATP, and they could be more sensitive to changes in ATP levels [30].

The inhibition of cell proliferation and the induction of apoptosis were significantly higher in Jurkat cells than in CRL-1991 cells, and it may occur due to the action of cAMP and the sensibility of each cell line to higher levels of cAMP. More than 2000 published articles have demonstrated the relation between cAMP and apoptosis or inhibition of cell proliferation, and it was reported that such effects would depend on the cell type. In this regard, the increase of intracellular cAMP is reported to be pro-apoptotic in leukemic cells and anti-apoptotic in normal hematopoietic cells as CRL-1991, being the reason why some cancer treatments use cAMP analogs, phosphodiesterase E inhibitors, and adenyl cyclase activators [31]. In addition, the most effective mechanism to increase intracellular cAMP levels is through MRP4 inhibition, and studies in S49 lymphoma cells have determined cAMP triggers apoptosis via PKA leading intrinsic, mitochondria-dependent mechanism [32]. Another factor regarding selective apoptosis is the selective involvement of the PKA isozymes I and II, where cAMP analogs selective for type I regulatory subunit inhibit natural killer-mediated cytotoxicity. However, expression of type II regulatory subunit modulates apoptosis of fibroblasts. Deregulation of the balance between these isozymes have been linked to various cancers, and the manipulations of such isozymes may increase or decrease cAMP-mediated apoptosis [31]. Concerning CRL-1991 cells, it was reported that the guanine nucleotide exchange factor Epac, also a second effector of cAMP an, mediates the anti-apoptotic effect once cAMP levels are increased [33]. In addition, the phosphorylation of dynamin-related protein 1 by PKA is reported to protect against apoptosis [34]. Such events may explain the lower sensitivity of CRL-1991 to 6-MP and Ceef1. The approaches focused on the inhibition of MRP4 to induce cAMP-mediated apoptosis shall be extensively studied in each cell type, to elucidate the mechanisms involved in the selectivity between cancer and normal cells.

## 5. Conclusions

The antiproliferative and apoptotic effect by Ceef1 was evaluated for the first time in Jurkat and CRL-1991 cells, as the first step to further evaluate its enhancing activity on the 6-MP chemotherapeutic drug. Once we determined that Ceef1 enhanced the activity of 6-MP, the next goal is determining the mechanisms involved in such additive cooperation. We are currently working on genetic engineering techniques and in silico experiments to better understand the architecture and function of MRP4 and the interaction with Ceef1 and other molecules, as basic research for gene therapy. We also conclude that the MRP4 specific inhibitor Ceef1 induces apoptosis *per se*, and it could be tested in vivo and in primary cell cultures to determine if they can be used in combined chemotherapy protocols.

## Figures and Tables

**Table 1:** Concentrations of both 6-MP and ceefourin-1 for the proliferation and viability assays.

	Concentration μM				
6-MP	0.1	1.0	5.0	10	50
Ceefourin-1	0.1	1.5	10	50	--

**Table 2:** Docking Scores and binding energies (ΔGs) of the MRP4 complexes.

Complex	Docking Score (kcal/mol)	ΔG (kcal/mol)
6-MP-MRP4	−4.79	−17.86
Ceefourin-1-MRP4	−7.63	−26.12

## Data Availability

Not applicable.
